# Combination Therapy of Infections Caused by Injection of Paint Using Medical Laser

**Published:** 2017-01

**Authors:** Shahrokh Attarian, Afsaneh Karami, Faezeh Ayatolahi

**Affiliations:** 1 Plastic Surgery, Zanjan University of Medical Sciences, Zanjan, Iran;; 2 Infectious disease specialists, Zanjan University of Medical Sciences, Zanjan, Iran;; 3 Laser Ward, Zanjan University of Medical Sciences, Zanjan, Iran

**Keywords:** Laser, Therapy, Infections

**DEAR EDITOR**

Doubtlessly, one of the most important developments in medical science has been the discovery of antibiotics and their use in the treatment of infectious diseases caused by the various bacteria. However, every day we witness the emergence of resistant strains of bacteria which their resistance power against the antibiotics are increasing; although scientists discover new antibiotics every year to supply the consumption market, antimicrobial resistance is a much faster trend and therefore it slows the healing process of many infectious diseases.^1^ In the modern medical technologies, it is tried to eliminate the germs and remove pus of the lesion, as well as draining out the infection site using alternative solutions rather than the use of antibiotics.^2^

Laser is a new technology which in recent years, it has found many applications in various scientific fields such as military and civilian industries as well as various branches of medical science. The action mechanism of laser energy is to absorb the light energy by the molecules in the tissue which causes the certain tissue molecules becoming warm, hot and ultimately destroys them; whereas laser is a mono-wavelength beam of light, it is able just to heat up and destroy specific structures.^3^ Some anti-inflammatory effects of laser were previously mentioned.^4^^-^^6^

On May 25^th^ 2013, a 27-year old woman visited the Emergency Department of Ayatolah Mousavi Hospital with the history of schizophrenia, drug use, and hyper-lipidemia. During the examination, it was found that the patient was injected the paint into the left forearm and right thigh last week which led to the edema and erythema at the injury site. Two days after the injection, purulent discharge was observed at the injury. She was hospitalized initially at the burn unit of the hospital, and then the patient was transferred to the infectious diseases unit of Valiasr Hospital due to the severe infection of soft tissue at the injury. Body temperature=8/36, HR=86, RR=19, BP=10/6.

Through the examination, erythematous lesions were observed with the necrotic center of 6-7 cm diameter having purulent discharge on the forearm to the elbow, and also the erythematous and edematous lesions containing scattered pustules in an undulation form were seen on the patient’s right thigh injury. The drugs, including clindamycin, vancomycin, ciprofloxacin and ryfampy have been prescribed for the treatment, as well as fluvoxamine, inderal, respridone, thiothixene, clonazepam, and pethidine according to the psychiatrist. Later, the arterial and venous Doppler ultrasound was performed on the injury site with following results. 

The venous blood flow in the auxiliary vein, brachial vein, and cephalic and bazilic proximal vein network of left elbow was within the normal range; the arterial blood flow and spectrum Doppler in the auxiliary and brachial arteries of the left upper limb was observed in the normal range. It was not possible to examine the radial and ulnar arteries due to the dressing on the left forearm, however, arterial profusion of all left hand fingers was observed natural. 

bone scan: (i) Bony lesion in the right knee region due to osteomy elitis, and (ii) there was infection in the soft tissue of the right thigh as well left lower arm. WBC=11000 (P=70%, L=18%, EO=4%, Mon=8%), Hb=10/7, PLT=65000, ESR=102, BUN=5/5, CR=0/6, U/A=normal, NA=142, K=4/2, BS=108, AST=107, ALT=27, ALP=335, LDH=338, Iron=52, TIBC=405, Retic=0/8, and Ferritin=736/4. Later: WBC=11000, HB=11/6, PLT=281000, ESR=70, and U/C=*E. coli*.

The major reason of the positive charge of lesion is the presence of metal cations and metalloids, such as iron of the hemoglobin in red blood cells, or calcium and other metals which exist in the basal membrane. It should be noted that the reactions between positively and negatively charged molecules plays an important role in antimicrobial defense, healing and tissue growth. Using laser leads the positive charge to be increased in the lesion site through influencing on metals, releasing the electron from valence shell (outermost electron-occupied shell in the atom), and transferring it to the amine circuit, hydroxyl or methyl and ethyl branches of proteins which contributes considerably in antimicrobial defense and healing; however, that effect is more effective in the wave lengths ranged 1064 nm to 532 nm, - i.e. out of the infra red wave length range, and in the biophotonic form. Note that, for the wave lengths of infra red, such as 10640 nm related to CO_2_ laser, the effects will be mainly biothermic and biomechanical with the action of debridement on the lesion or increasing the vessels surrounding the edge of lesion in a way that influences on the healing process. The above mentioned characteristics lead the CO_2_ laser to become one of the most appropriate solutions for the supplement treatment accompanied with antibiotics in some of the infectious diseases.^7^^-^^9^

According to the surgeon at the center, it was more likely to be occurred necrotisin fasciitis, and the surgeon recommended the debridement. After the patient examination, the orthopedist diagnosed the amputation; while, after the hematological evaluation, it was recommended to perform a control test in relation to the anemia and thrombocytopenia according to the normal blood lamella. During the patient examination by the plastic surgeon, there was no fever symptom observed, while septicity and pus of the lesion was evident; hence, the plastic surgeon recommended the CO_2_ and Ar laser therapy as the treatment of septicity and pus.

The laser treatment was followed by the treatment has been performed in three below stages: (i) Discharging the dye from the lesion; (ii) Draining the infectious site; and (iii) Filing the lesion site. The abscess stage was performed in a therapy session in the operation room under the sedation. The CO_2_ surgical laser was used to discharge the paint and pus from the abscess and prepare the lesion site for the remaining treatment stages. The technical specifications and light of utilized laser device are as follows: Wave Length: 10640 nm, Super Plus, and Continuous. 15 watt $\left\{\begin{array}{c} 1000\mathrm{ms on time}\\ 50\mathrm{ms off time} \end{array}\right.$

Neutralization of the toxic metals of paint and also destroying microbes stage was performed in two sessions on the out-patient basis within one week interval utilizing the laser device with below technical specifications: Q Switch 

532 nm501064 nm 2000 MJ, 6 HTZ, 400mjcm2s

At the fourth week, and after the completion of treatment, the lesion was dressed and the treatment was considered successful. For treatment of complex and complicated lesions, it is possible to utilize the laser technology in accordance with the standard protocols, especially photodynamic therapy as the supplement for the antibiotic therapies and healing the resistant cases. 

## CONFLICT OF INTEREST

The authors declare no conflict of interest.
